# Prognostic Significance of MET Amplification and Expression in Gastric Cancer: A Systematic Review with Meta-Analysis

**DOI:** 10.1371/journal.pone.0084502

**Published:** 2014-01-08

**Authors:** Zhi Peng, Yan Zhu, Qianqian Wang, Jing Gao, Yilin Li, Yanyan Li, Sai Ge, Lin Shen

**Affiliations:** 1 Department of Gastrointestinal Oncology, Key laboratory of Carcinogenesis and Translational Research (Ministry of Education), Peking University Cancer Hospital & Institute, Beijing, China; 2 Department of Epidemiology, Beijing Jishuitan Hospital, the Fourth Clinical Medical College of Peking University, Beijing, China; Centro di Riferimento Oncologico, IRCCS National Cancer Institute, Italy

## Abstract

**Background and Aims:**

MET, the hepatocyte growth factor receptor, is a receptor tyrosine kinase overexpressed and activated in a subset of gastric cancer. Several studies investigated the relationship between MET amplification and expression with the clinical outcome in patients with gastric cancer, but yielded conflicting results. We performed a systematic review and meta-analysis to determine the influence of MET amplification and expression on prognosis in gastric cancer.

**Methods:**

MEDLINE and EMBASE were searched for studies that explored the association between MET amplification and expression with survival in patients with gastric cancer up to 1 April, 2013. Data of individual hazard ratios (HRs) and 95% confidence intervals (CIs) for meta-analyses were extracted from the publications and combined in pooled HRs.

**Results:**

Fourteen studies involving 2,258 patients with gastric cancer were included. It was suggested that MET overexpression had an unfavorable impact on survival of patients with gastric cancer, with HRs (95% CIs) of 2.57 (95% CI: 1.97–3.35) overall, 2.82 (95% CI: 1.86–4.27) among studies using amplification for measure scale of MET and 2.42 (95% CI: 1.66–3.54) for expression. The magnitude of association was reduced whereas remained statistically significant in high quality studies or in larger sample size studies and corresponding HRs were 2.18(1.76, 2.70) and 2.35(1.93, 2.87), respectively, without significant heterogeneity.

**Conclusion:**

The findings from present study indicated that higher MET gene amplification and expression in gastric cancer was an indicator of poor prognosis.

## Introduction

Each year, it is estimated that nearly one million new cases and over 700,000 deaths from stomach cancer occurred, accounting for 8% of the total cancer cases and 10% of total cancer deaths [1]. Although the incidence of gastric cancer has decreased substantially over the recent few decades in most parts of the world, but it is still one of the most common cancer types worldwide [2]. What is more, overall survival remains poor, especially for advanced gastric cancer, and no established global standard for treatment has been set. Discovering new therapies which target specific genetic alterations arguably provide a more personalized treatment for gastric cancer [3].

The discovery of molecular biological prognostic factors could provide a more accurate prediction of clinical outcome and may also reveal novel predictive factors and therapeutic targets [4]. The most frequently studied putative molecular biological prognostic factors in gastric cancer are human epidermal growth factor receptor 2 (HER2/neu), epidermal growth factor receptor (EGFR), vascular endothelial growth factor receptor (VEGFR), cyclooxygenase 2, hepatocyte growth factor receptor (HGFR/MET) and etc. Trastuzumab, a monoclonal antibody targeting HER2, has been successfully approved as the first molecularly targeted drug against patients with HER2 positive gastric cancer [5].

MET, is a proto-oncogene that encodes a protein also known as HGFR. The MET tyrosine kinase receptor promotes tissue remodeling, which underlies developmental morphogenesis, wound repair, organ homeostasis and cancer metastasis, by integrating growth, survival and migration cues in response to environmental stimuli or cell-autonomous perturbations [6]. Moreover, MET has been indicated as an attractive target for cancer therapy. Agents targeting MET pathway such as inhibitors or monoclonal antibody have been introduced into the clinical application [7].

Many retrospective studies have evaluated whether overexpression of MET is a prognostic factor for survival in patients with gastric cancer. However, the results of these studies are inconclusive. Therefore, a systematic review and meta-analysis was conducted to assess the prognostic value of MET overexpression on survival in patients with gastric cancer.

## Materials and Methods

### Search strategy and selection criteria

A systematic review of published work was conducted according to the Preferred Reporting Items for Systematic Review and Meta-Analyses guidelines [8]. Electronic searches was performed of the English-language literatures on MET expression and amplification of gastric cancer in PubMed, EMBASE, and The Cochrane Library using the combined text words “stomach neoplasms” and proto-oncogene proteins MET or MET or Hepatocyte growth factor receptor or HGF Receptor or Scatter factor Receptor or Proto-Oncogene proteins, met. The latest search was undertaken in 1 April, 2012. We also manually screened the reference lists of the retrieved articles to identify other relevant publications.

Translational studies eligible for inclusion in this meta-analysis met the following criteria: (1) measure MET amplification or expression in the gastric cancer tissue with Silver In Situ Hybridization (CISH) or immunohistochemistry (IHC) or reverse transcription-polymerase chain reaction (RT-PCR) or real-time polymerase chain Reaction (qPCR) and etc; (2) provide data of a hazard ratio (HR) and 95% confidence interval (CI) or sufficient data to calculate HR and 95% CI. When there were more than two articles using the overlapped populations, the most recent publication was included. Review articles, case reports, experimental studies and studies that did not report outcomes were excluded. Unpublished data from conference abstracts were excluded either.

### Data Extraction

Data was extracted by two investigators (Peng and Zhu) independently using a standard protocol. Any discrepancies were resolved by discussion and consensus. The following data elements were extracted from each study: first author, year of publication, time of collection, race, No. of patient (male/female), tumor stage, technique of detection, classification of MET positive, positive rate, hazard ratios (HRs) and 95% confidence intervals (CIs).

HRs and 95% CIs [9] were used to combine as the effective value. If both the crude and adjusted HRs and their 95% CIs were reported in the articles, we used the former ones. When these variables were not given explicitly, statistical method developed by Parmar et al [10] was used to indirectly estimate hazard ratios from Cox regression analyses and *P* values from log-rank tests.

### Quality Assessment

Study quality was assessed independently by two researchers (Peng and Zhu) by means of a predefined form by De Graeff [11] and MJM Gooden [12]. Reporting recommendations for tumor marker prognostic studies (REMARK) was adapted from the work of Hayes [13] and McShane [14]. Briefly, the following criteria were included: whether (1) the study reported inclusion and exclusion criteria; (2) study data were prospectively or retrospectively gathered; (3) patients and tumor characteristics were sufficiently described; (4) the method used to measure MET amplification or expression was sufficiently described; (5) the start point and endpoint of the study was provided; (6) the follow-up time of patients in the study was described; (7) the study reported how many patients were lost to follow-up and the percentage should be below 10%. Studies with a total score of 8 were considered to show the highest study quality, whereas a zero score indicated the lowest study quality.

### Statistical Analysis

The effect of associations was estimated as HR with the corresponding 95% CI. Meta-analysis is generally carried out with the natural logarithm of the HR and its standard error, to make the range of HRs symmetrical. After log transformation, a HR of 0 becomes minus infinity, a HR of 1 becomes 0, and a HR of infinity remains infinity. Firstly, fixed-effect model was used for calculating pooled HRs. If there were significant heterogeneity across studies, random-effect model was selected. The existence of heterogeneity between studies was evaluated using the Dersimonian and Laird's Q test [15]. *I* ^2^ was used to quantify heterogeneity; this measure describes the percentage of the observed between-study variability attributable to heterogeneity rather than chance. *I* ^2^ takes values between 0% and 100%. An *I* ^2^ value >50% was considered to represent substantial heterogeneity between studies [16].

By convention, an observed HR >1 implied a worse survival for the group with positive MET expression. This impact of MET on survival was considered as statistically significant if the 95% CI for the overall HR did not overlap 1.

In this meta-analysis, score over 5 was defined as high quality studies and others were low quality studies accordingly. Studies were also classified into 2 groups by sample size (<100 and ≥100). Meta-regression analyses considered quality score and sample size (continuous variables). We also performed a cumulative meta-analysis to assess the evolution of the observed effects over time.

Publication bias was evaluated using inverted funnel plot and Egger's test [17]. If there was publication bias, the nonparametric “trim and fill” method was used to adjust our analysis. All analyses were carried out using Stata software (version 11.0). All *P* values were two-sided and the significance level was 0.05.

## Results

### Study selection and characteristics

**Figure 1:**
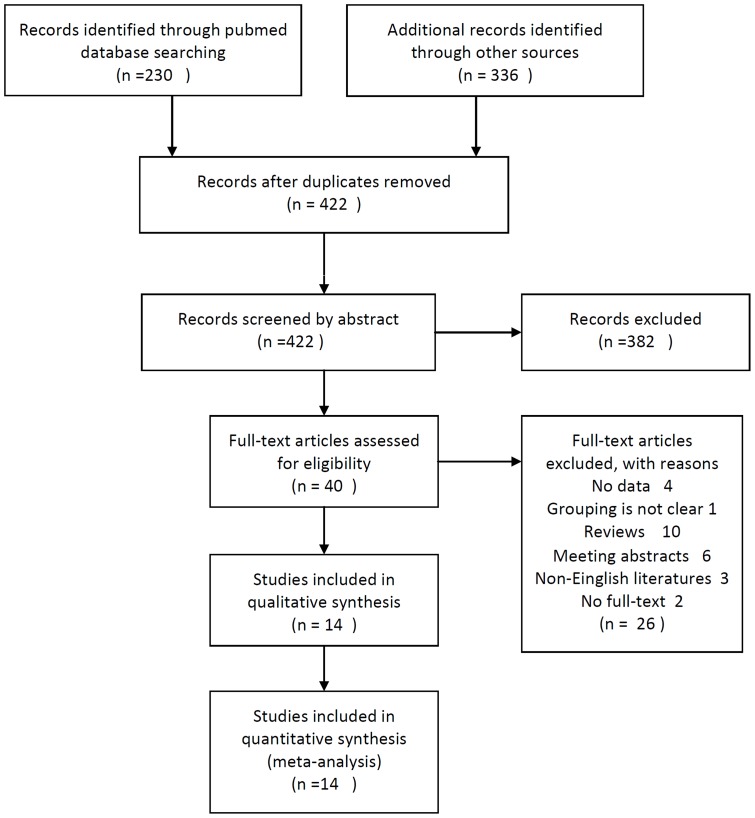
Flow chart of the eligible studies. Flow chart of the eligible studies for the meta-analysis of c-Met overexpression and prognosis of gastric cancer, with specifications of reasons.

Totally 422 articles were identified from electronic databases, of which 40 studies potentially related to our issue. Finally, fourteen articles were included for the meta-analysis in accordance with the selection criteria (Fig. 1) [18]–[31]. These studies concerned different cohorts of patients published between 1998 and 2012. The total number of included patients was 2,258, ranging from 35 to 544 patients per study. There were 5 prospective studies[19], [25], [26], [29], [30] and 9 retrospective studies[18], [20]–[24], [28], [31], [32]. Table 1 summarizes characteristics of all inclusive studies. Eleven studies concerned patients with all stages (I–IV), 2 for II-IV stages [23], [25] and 1 for II-III stages [20]. Among the 14 studies, 10 studies (1,851 patients, 82%) were performed in Asian populations, and the remaining 4 studies (407 patients, 18%) were in Western populations [20], [26], [30], [31].

**Table 1:** Main characteristics of clinical trials included in meta-analysis.

First author	Year of Publication	Time of collection	Race	No. of patient(male/female)	Stage	Technique	Expression or amplification	Classification of c-MET positive	positive rate	HR estimation	Study quality score
Tsugawaa	1998	1986–1991	Asian	70(NA)	I–IV	Slot Blot Hybridization Analysis	amplification	ratio>2	10%(7/70)	survival curves	4
Toiyama	2011	2000–2005	Asian	100(73/27)	I–IV	RT-PCR	expression	NA	24%(24/100)	survival curves	5
Graziano	2011	1998–2007	Caucasian	216(124/92)	II–III	qPCR	amplification	GCN≥5	10%(21/216)	HR+CI 95%	7
Li	2012	2004–2007	Asian	114(72/42)	I–IV	IHC	expression	score≥4	82.4% (94/114)	HR+CI 95%	5
Shi	2012	1999–2006	Asian	128(101/27)	I–IV	qPCR	amplification	GCN≥4	30% (39/128)	HR+CI 95%	5
Lee	2011	NA	Asian	472(316/156)	II–IV	qPCR	amplification	GCN≥4	21.2%(100/472)	HR+CI 95%	6
Lee	2012	2004	Asian	381(274/107)	II–IV	SISH	amplification	High polysomy and GA	19.4%(74/381)	HR+CI 95%	6
Huang	2001	1997–1998	Asian	45(33/12)	I–IV	IHC	expression	≥5% of tumor cells	71.1%(32/45)	RR+CI 95%	6
Drebber	2007	NA	Caucasian	114(67/47)	I–IV	IHC	expression	>30% of tumor cells	73.7%(84/114)	HR+CI 95%	7
Nakajima(1)	1999	1991–1996	Asian	128(94/34)	I–IV	IHC	expression	>5% of tumor cells	46.1%(59/128)	survival curves	4
Nakajima(2)	1999	1991–1996	Asian	128(94/34)	I–IV	Southern blot analysis	amplification	Ratio>2	10.2%(13/128)	survival curves	4
Taniguchi	1998	1990–1995	Asian	102(NA)	I–IV	IHC	expression	>30% of tumor cells	42%(43/102)	survival curves	4
Zhao	2011	2003–2007	Asian	182(121/61)	I–IV	IHC	expression	score≥3	65.9%(120/182)	HR+CI 95%	6
Kubicka	2001	NA	Caucasian	42(NA)	I–IV	IHC	expression	>10% of tumor cells	26%(11/42)	survival curves	4
Catenacci(1)	2011	2002–2008	Caucasian	35(NA)	I–IV	IHC	expression	NA	43%(15/35)	HR+survival curves	3
Catenacci(2)	2011	2002–2008	Caucasian	45(NA)	I–IV	qPCR	amplification	GCN≥7	8.3%(3/36)	HR+survival curves	3

NA: Not Available; HR: Hazard Ratio; RR: Relative Ratio; CI: Confidence Interval; IHC: Immunohistochemistry; GCN: Gene Copy Number; SISH: Silver In Situ Hybridization; RT-PCR: Reverse Transcription-Polymerase Chain Reaction; qPCR: real-time Polymerase Chain Reaction.

Methods to determine MET status included IHC (n = 8) [21], [25]–[31], qPCR (n = 4) [20], [22], [23], [31], RT-PCR (n = 1) [19], SISH (n = 1) [24], southern blot (n = 1) [27] and slot blot hybridization analysis (n = 1) [18]. Positive rate of MET amplification ranged from 8.3 to 82.4% among studies. Nine studies used the method of MET expression and seven studies used gene amplification to explore the relationship between MET and prognosis of patients with gastric cancer. Two studies used both methods for determining MET status. Nakajima's study [27] performed IHC and Southern blot analysis separately in the same population, whereas Catenacci's study [31] used different population for IHC and qPCR test. Both of them were separated into 2 independent trials for meta-analysis.

Eleven of the 14 studies identified MET overexpression as a poor prognostic factor for survival whereas no report indicated it was a good prognostic factor. Seven of these 14 papers reported HRs and 95% CIs while one other study provided RR and 95% CI [25], five with survival curves and one provide HR point estimate plus survival curves [31].

The 14 studies had a median quality score of 5 out of 8 (range 3–7) with published in journals with a mean impact factor of 4.29 (range: 1.835–18.372).

### Meta-analysis

**Figure 2:**
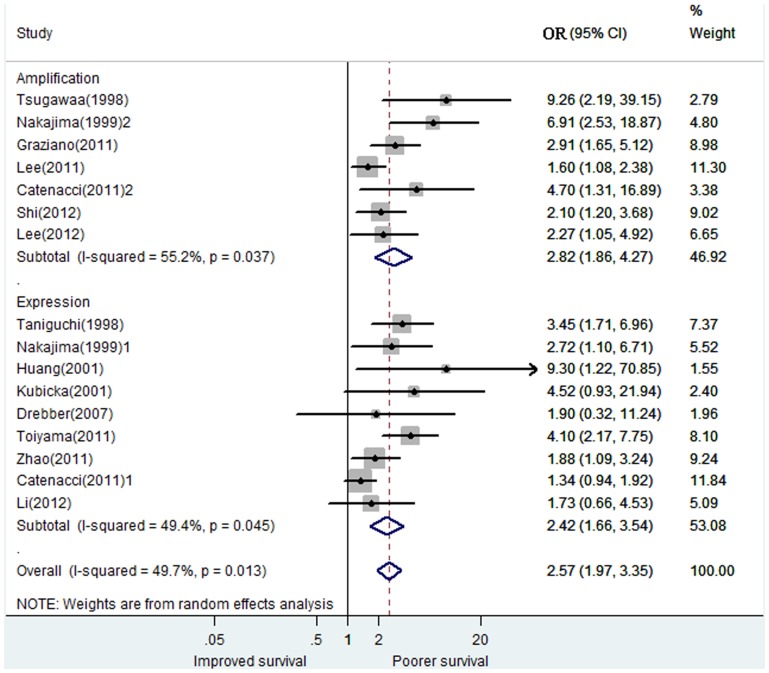
Forest Plot of Results of the Prognostic Value of MET Over-expression. Influence of MET amplification or expression on prognosis in all patients with gastric cancer. Weights are from random-effects analysis. Squares indicate the point estimates of the effect of disease (odds ratio) and diamonds, the summary estimate from the pooled studies; 95% confidence intervals are indicated by horizontal bars and shown in parentheses.

**Figure 3:**
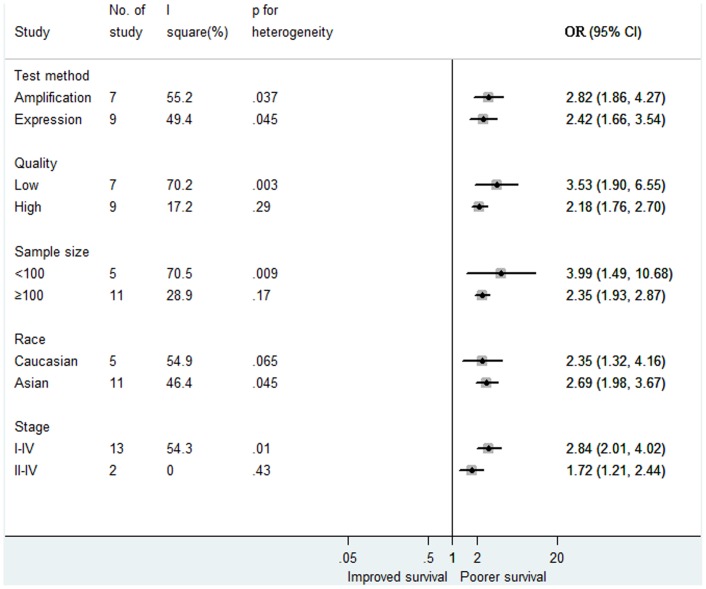
Effect of MET overexpression on gastric cancer by prespecified study characteristics in different subgroups. Weights are from random-effects analysis. Squares indicate the point estimates of the effect of disease (odds ratio) and diamonds, the summary estimate from the pooled studies; 95% confidence intervals are indicated by horizontal bars and shown in parentheses.

The combined HRs for 14 studies evaluating MET overexpression on overall survival was 2.57 (95% CI: 1.97–3.35), suggesting that MET overexpression was an indicator of poor prognosis for gastric cancer (Fig. 2). However, significant heterogeneity was observed among the studies (*I* ^2^ = 49.7%, *P* = 0.013). For subgroup analyses, there was no significant heterogeneity among the studies with high quality (quality score≥5) or with large sample size (sample size≥100), corresponding with combined HRs of 2.18(1.76, 2.70) and 2.35(1.93, 2.87), respectively. For subgroup analyses based on race and tumor stage, all the results are suggesting that MET overexpression had a significant poor impact on survival (Fig. 3).

Influence analysis showed that removal any research would not cause a significant change in the results. In a cumulative meta-analysis, the effect of MET overexpression was not changed overtime (supplement Fig. 1). A sensitivity analysis excluding the studies of which the HRs (95% CI) was estimated from the survival curves did not alter the associations (HR = 1.83, 95% CI: 1.51–2.21, *P*<0.001).

### Publication bias

For publication bias estimating, we observed visually and statistically significant asymmetry according to the inverted funnel plot (supplement Fig. 2) and Egger's test in all analyses (data not shown). However, after adjustment for the effect of publication by Tweedie's trim and fill method, the magnitude of the association between MET and the prognosis of the patients with gastric cancer was not materially changed, with HR of 1.88(1.40, 2.51). We also tested the publication bias among the large sample studies or among the high quality studies and no significant publication bias were observed.

## Discussion

Identification of prognostic factors allows the definition of high-risk groups of patients for whom specific therapy might be necessary, or stratification has to be performed in controlled trials [33]. In this study, we present a pooled estimate of the prognostic value of MET amplification and expression in gastric cancer. The present systematic review and meta-analysis suggests that MET overexpression is an indicator of poor prognosis for patients with gastric cancer. Thus, inhibiting MET pathway might be an effective treatment for gastric cancer.

The biological role of MET can explain its poor prognosis. There are some evidence that MET is a key driver of oncogenic transformation in a defined subset of cancers. The prognosis of other cancer types such as non-small cell lung cancer, colorectal cancer, ovarian cancer, breast cancer were also reported associated with the high levels of MET and/or HGF expression [34]. Moreover, several clinical trials have shown clinical benefits from inhibition of the pathway of MET in patients with gastric cancer. Interruption of the signaling pathways for MET can be achieved using antibodies (Rilotumumab and MetMAb) or small-molecule, orally-active, tyrosine kinase inhibitors (tivantinib) [7]. Both approaches have been demonstrated effective and may provide a future treatment for advanced gastric cancer. Our result will give us a clue how to select suitable patients with gastric cancer for anti-MET therapy, which will more suitable and cost-effective.

This systematic review with meta-analysis was complicated by heterogeneity issues. We found significant heterogeneity among overall 14 studies and subgroup of amplification and expression. When the analysis was limited to high quality subgroup and large sample size subgroup, heterogeneity was not detected. The source of heterogeneity might be resulted from low quality study.

There are some other confounders must be mentioned in this meta-analysis. Meta-analysis of prognostic literature is associated with a number of inherent limitations such as retrospective study design, the availability and adequacy of corresponding clinico-pathological data, and the general lack of multivariable survival data [35].

Publication bias is a common concern for meta-analysis. Full text of two papers cannot be obtained [36], [37]. Articles published using other languages such as Venezuela [38] and Chinese [32], [39] were excluded either. However, all of these five studies are reported poor prognosis when MET expression is high. Therefore, overall effect will not change when these papers were included. Four eligible trials had to be excluded from the meta-analysis because they did not provide sufficient data on survival [40]–[43]. Among the four excluded studies, two of these studies were not statistically significant [40], [41]. It is known that this type of study is less frequently published or, if they are, with less detailed results, making them less assessable. In present study, trim and fill method was used for sensitivity analysis of publication bias and the results was not materially changed.

The techniques used to detect MET amplification and expression might also be considered. The articles involved in this meta-analysis spanned 15 years from 1998 to 2012, and hence various laboratory assays were used to determine HER2 gene amplification and expression. These differences in methodology can be seen from the wide range of MET amplification (8.3 to 21.2%) and expression positivity (26 to 82.4%) in this study. Because of the fact that an optimal threshold has not been defined, the cut-off defining a gastric cancer with MET expression and gene amplification is arbitrary, which might produce bias. There are few papers comparing the consistency between MET gene amplification and protein expression, thus no consensus until now. We can speculate that the proportion of gene amplification is lower than positive protein expression from these included articles. In LEE's study, both HR of amplification and protein expression are positive, which has good correlation between high protein expression and gene amplification [24]. But no correlation was found between high MET protein expression and MET gene amplification in Yelena's study [44]. Large population based control study should be performed to confirm the result. Standardization of FISH and IHC testing is therefore essential. Despite these differences, results from subgroup analysis related to test method (amplification or expression) were similar to the overall analysis.

In conclusion, the present meta-analysis is the first study to systematically estimate the association between MET overexpression and gastric cancer survival. The present systematic review and meta-analysis shows that MET overexpression and gene amplification was an indicator of poor prognosis in patients with gastric cancer. In order to become a useful prognostic factor in the clinical practice, a standardization of the FISH and IHC techniques are needed, particularly concerning the positivity threshold. In addition, the present results need to be confirmed by an adequately well designed prospective study with an appropriate multivariate analysis taking into account the classical well-defined prognostic factors for survival in gastric cancer patients.

## Supporting Information

Figure S1 **Influence analysis for the effect of MET overexpression.**
(DOCX)

Figure S2 **Funnel plots for the publication bias estimating.**
(DOCX)

Checklist S1 **PRISMA checklist.**
(DOC)
