# Supplementary material for: Prognostic Significance of MET Amplification and Expression in Gastric Cancer: A Systematic Review with Meta-Analysis
Source: PLoS One. 2014 Jan 8;9(1):e84502. doi: 10.1371/journal.pone.0084502 (PMC3885582; doi:10.1371/journal.pone.0084502)
Supplement: Figure S1 — Influence analysis for the effect of MET overexpression. (DOCX) [file pone.0084502.s001.docx]

**Figure S1.**


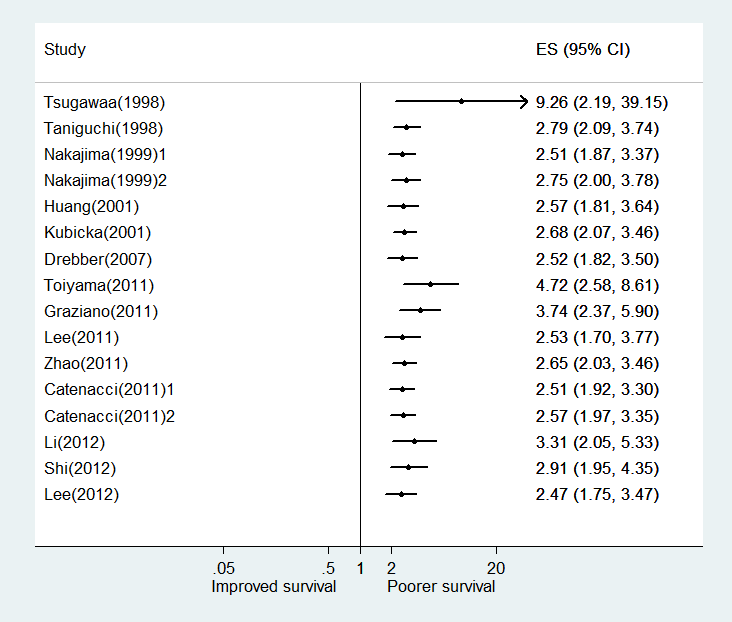


Influence analysis for the effect of MET overexpression was not changed overtime. Circles represent the pooled odds ratios (OR) after excluding one study at a time. The dashed line represents 95% confidence intervals.
