# Supplementary material for: Prognostic Significance of MET Amplification and Expression in Gastric Cancer: A Systematic Review with Meta-Analysis
Source: PLoS One. 2014 Jan 8;9(1):e84502. doi: 10.1371/journal.pone.0084502 (PMC3885582; doi:10.1371/journal.pone.0084502)
Supplement: Figure S2 — Funnel plots for the publication bias estimating. (DOCX) [file pone.0084502.s002.docx]

**Figure S2.**


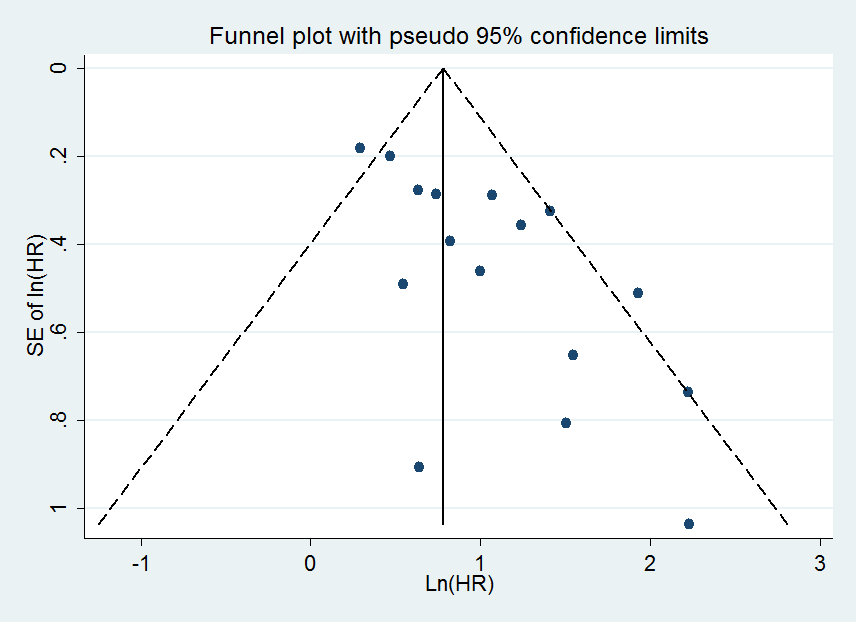


Funnel plots for the publication bias estimating. The dashed line represents 95% confidence intervals. Circles represent individual studies.
